# Integrated analysis of the expression profiles of the lncRNA-miRNA-mRNA ceRNA network in granulosa and cumulus cells from yak ovaries

**DOI:** 10.1186/s12864-022-08848-3

**Published:** 2022-09-03

**Authors:** Ling Zhao, Yangyang Pan, Meng Wang, Junqian Wang, Yaying Wang, Xiaohong Han, Jinglei Wang, Tongxiang Zhang, Tian Zhao, Honghong He, Yan Cui, Sijiu Yu

**Affiliations:** 1 College of Veterinary Medicine, Gansu Agricultural University, Lanzhou, 730070 China; 2 Gansu Province Livestock Embryo Engineering Research Center, College of Veterinary Medicine, Lanzhou, 730070 China

**Keywords:** Granulosa cells, Cumulus cells, LncRNA, miRNA, ceRNA network

## Abstract

**Background:**

Growing oocytes acquire the ability to mature through two-way communication between gametes and surrounding somatic cumulus cells (CCs). Granulosa cells (GCs) support oocyte growth, regulate meiosis progression, and modulate global oocyte transcription activity. However, the proliferation and differentiation of the yak ovary in GCs and CCs remain unclear. To characterize the important roles of long non-coding RNA, (lncRNA), microRNA (miRNA), and messenger RNA (mRNA), whole-transcriptome analysis was performed. Real-time quantitative fluorescence PCR was performed to verify the selected RNA sequences.

**Results:**

Important gene ontology terms and Kyoto Encyclopedia of Genes and Genomes pathways related to differentiation and oocyte development were identified for the target genes of differentially expressed lncRNAs, miRNAs, and mRNAs. In total,6223 mRNAs (2197 upregulated, 4026 downregulated), 643 lncRNAs (204 upregulated, 479 downregulated), and 559 miRNAs (311 upregulated, 248 downregulated) were significantly altered between the two groups. Target genes involved in cell adhesion, cell differentiation, regulation of developmental processes, cell proliferation, embryo development, signal transduction, apoptosis, and aromatic compound biosynthetic processes were significantly enriched. These RNAs were involved in ECM-receptor interaction, MAPK signaling, Hippo signaling, PI3K-Akt signaling, cell cycle, cell adhesion, leukocyte trans-endothelial migration, and actin cytoskeleton regulation.

**Conclusions:**

A comprehensive analysis of the co-expression network of competing endogenous RNAs (ceRNAs) will facilitate the understanding of the process of granulosa cell proliferation and differentiation and offer a theoretical basis for the development of oocytes.

**Supplementary Information:**

The online version contains supplementary material available at 10.1186/s12864-022-08848-3.

## Background

In mammals, folliculogenesis and oogenesis occur in parallel within the ovary [1]. The ovarian follicle consists of an oocyte surrounded by theca and granulosa cells (GCs) involved in ovulation, fertilization, implantation, and embryo growth [2]. GCs preserve and breed oocytes and secrete steroid hormones, such as estrogen and progesterone, to provide a crucial microenvironment for follicular growth [3]. Proliferation and differentiation of GCs is fundamental to the development of the follicle and the oocyte, to ovulation, and to luteinization, while apoptotic cell death performs the mechanism of ovarian follicle atresia. GCs are considered to maintain ovarian function. Growing oocytes in the ovary acquires the ability to mature through a two-way communication between the gamete and the surrounding somatic cumulus cells (CCs) [4–6]. Oocytes secrete a variety of growth factors to promote CC differentiation and proliferation [7]. These secreted molecules are mainly responsible for maintaining different stages of CCs and preventing their differentiation into membrane or granular cells [8].

Molecular replacement is the connection between the oocyte membrane and CCs through gap junctions [9]. Oocyte-granulosa cell communication is essential for the normal growth and development of both oocytes and follicles. Communication occurs via a bidirectional communication axis involving paracrine signaling and the gap-junctional exchange of small regulatory molecules [10]. The genetic and transcriptional characteristics of the cumulus-oocyte complex (COC) are reflected in the developmental potential for successful fertilization and embryonic development. However, little is known about the relationship between genes expressed in oocytes and CCs [11]. Studies that analyze the transcriptome of GCs and CCs may be a powerful tool to improve our knowledge of the pathways involved in oocyte development. Microarray technology has enabled the discovery of many ontological groups that are associated with oocyte development, CC and GC proliferation, and apoptosis.

This study aims to explore the markers of GCs and CCs that participate in the development of oocytes and affect cell proliferation, migration, and apoptosis. Yak GCs and CCs were cultured in vitro. Transcriptome changes in granulosa and cumulus cell genes are involved in regulating cell migration, cell proliferation, and programmed cell death. Genes that were significantly different in the granulosa and cumulus cell types were analyzed. Our results provide evidence on the proliferation and differentiation of GCs and CCs and can identify genes related to oocyte development.

A new mechanism of interaction between non-coding RNA and mRNA has been proposed, in which long non-coding RNAs (lncRNAs), messenger RNAs (mRNAs), and micro RNAs (miRNAs) are involved [12]. LncRNAs play a key role in many biological processes, including cell cycle regulation, epigenetic changes, and dose compensation [13]. Studies have shown that lncRNAs can also be used as competitive endogenous RNAs (ceRNAs), which act as miRNA sponges, and participate in regulating the expression of target genes [14, 15]. Generally, miRNAs function as part of ribonucleoprotein complexes, miRISCs (miRNA-induced silencing complexes), with miRNAs base-pairing to partially complementary sequences in the 3′ UTRs of target mRNAs. miRNAs inhibit protein synthesis by repressing translation and/or by bringing about deadenylation and subsequent degradation of mRNA targets [16]..LncRNAs and miRNAs play important roles in regulating ovarian maturation, ovarian cell developmental processes and hormone secretion [17–19]. In regulatory ceRNA networks, lncRNAs and miRNAs coordinate to integrate and regulate various aspects of ovarian tissue development. Although lncRNAs do not encode proteins, they have been shown to have transcriptional and post-transcriptional effects on gene function, because they usually participate in different physiological processes [20].

Therefore, the integration of lncRNAs and miRNAs can be used to study the regulatory mechanisms underlying ovarian growth at the molecular level [17]. To investigate the potential role of process granulosa cell proliferation and differentiation and to build miRNA, mRNA and lncRNA interaction networks in yak, we performed RNA-seq to identify genome-wide differentially expressed genes (DEGs), miRNAs and lncRNAs in each comparison. Gene Ontology (GO) [21] and Kyoto Encyclopedia of Genes and Genomes (KEGG) [22] enrichment analyses of DEGs and target transcripts of miRNAs, lncRNAs were conducted. These data will help to further our understanding of this mechanism on the influence of oocyte development.

## Methods

### Sample collection

Yak ovary samples were obtained from a local commercial slaughterhouse in Xining City, Qinghai Province, China. After learning about the sanitation and related conditions of the slaughterhouse, we entered to collect the yak ovaries. After the yak was slaughtered, the estrus ovaries containing antral follicles were picked, washed with sterile normal saline, stored at 4 °C, and quickly returned to the laboratory. A total of 477 samples were collected, of which 150 were used to collect GCs, and 327 were used to collect CCs. GCs and CCs were cultured in vitro and samples of GCs and CCs were harvested for RNA extraction, added into an Eppendorf tube without RNase, and immediately stored in liquid nitrogen. All samples were stored at − 80 °C until analysis.

### In vitro culture of yak granulosa and cumulus cells

The obtained follicular fluid was injected into a 15 mL centrifuge tube containing 2 mL of PBS. The follicular fluid was gently pipetted and mixed at 1000 rpm for 5 min. The supernatant was discarded, washed with 5 mL of DMEM/F12 and 10% FBS into a single suspension, and the trypan blue exclusion method was used. Next, we checked cell viability. The remaining cell fluid was washed again by centrifugation. The culture medium was discarded, an appropriate amount of cell culture medium was added, the cell density was adjusted to 5 × 10^5^, and inoculated in a cell flask or petri dish. Then, the petri dish was placed in a 5% CO2, 37 °C incubator for culture. The cells were observed under an inverted phase contrast microscope and the growth situation was recorded by photographing (Olympus CK41, Tokyo, Japan).

### LncRNA and mRNA sequencing and data processing

**Fig. 1:**
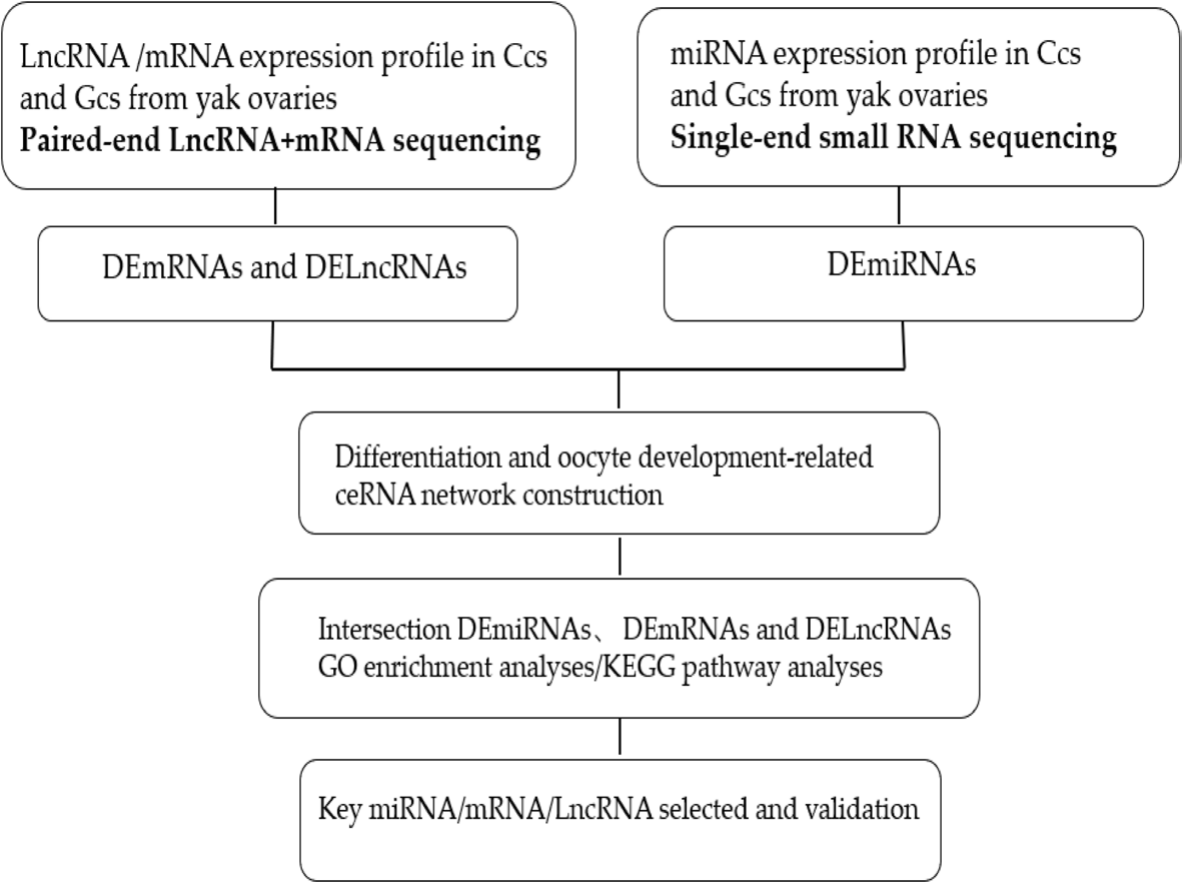
Workflow to identify the ceRNA-networks involved in granulosa and cumulus cells from yak ovaries

Total RNA was extracted using a TRIzol reagent kit (Invitrogen, Carlsbad, CA, USA) according to the manufacturer’s protocol. RNA quality was assessed using an Agilent 2100 Bioanalyzer (Agilent Technologies, Palo Alto, CA, USA) and verified by RNase-free agarose gel electrophoresis. Ribosomal RNAs (rRNAs) were then removed to retain mRNAs and lncRNAs using the epicenter RiboZero TM reagent. The remaining RNAs were randomly fragmented into short fragments that were used for cDNA synthesis. We performed library construction using a strand-specific method according to the following steps: firststrand cDNA was synthesized using random hexamers with rRNA-depleted RNA as a template. dNTPs, RNase H, and DNA polymerase I were used for second-strand synthesis. The cDNA was purified using AMPure XP beads. Next, cDNA fragment purification, end repair, poly(A) addition, and Illumina sequencing adapter ligation were carried out using the QIAquick PCR extraction kit. Then UNG (Uracil-N-Glycosylase) was used to digest the second-strand cDNA. cDNA fragments were size-selected by agarose gel electrophoresis, PCR-amplified, and sequenced using Illumina HiSeqTM 4000 by Gene Denovo Biotechnology Co. (Guangzhou, China). The applied workflow is shown in (Fig. 1).

Raw reads were first quality filtered by Fastp (V0.18.0) [23] to remove reads containing less than 10% unknown nucleotides (N),low-quality reads (> 50% bases, qvalue ≤20), and generate clean reads. All downstream analyses were performed based on high quality cleaning data. Bowtie2 (V2.2.8) [24] was used to map reads to a ribosomal RNA (rRNA) database. Clean, paired-end reads were mapped to the reference genome using HISAT2(V2.1.0) [25] with the significance of differentially expressed lncRNAs and mRNAs analyzed using the R package DESeq2 [26], *p*-values < 0.05, and fold-change > 2 being the filtering criteria to identify significant DEGs. Mapped reads from each library were assembled by StringTie (V1.3.4) [27]. Differential expression pattern clustering was then performed using the Gene Denovo platform. Expression levels of lncRNA and mRNA were normalized using FPKM (fragment of transcript per million mapped reads per kilobase). The CNCI [28], CPC [29], PFAM [30] and CPAT [31] were used to analyze the coding potential of the transcripts. The assembled transcripts, without the coding potential of their overlap, were the candidate set of lncRNAs. The reference genome and gene model annotation files of the yak (BosGru v2.0, https://www.ncbi.nlm.nih.gov/assembly/GCF_000298355.1/) were downloaded directly from the genome website.

### MiRNA library construction

Sequencing the RNA molecules in a size range of 18–30 nt were enriched using polyacrylamide.

gel electrophoresis (PAGE). Then, the 3′ adapters were added and the 36–44 nt RNAs were enriched. The 5′ adapters were then ligated to the RNAs. The ligation products were reverse-transcribed by PCR amplification and the 140–160 bp size PCR products were enriched to generate a cDNA library. To obtain a clean label, we filtered the original reading by:

Removing low quality reads;

removing reads without 3′ adapters;

removing reads containing 5’adapters;

removing reads containing ploy A in small RNA fragments;

removing reads shorter than 18 nt; and.

removing reads containing 3′ and 5′ adapters, but with no small RNA fragments between them.

All clean tags were aligned with the reference genome and then searched against the miRBase database [32] (Release 22) to identify known yak miRNAs. After excluding the reads mapped to known miRNA/ncRNA/repeat regions/mRNA regions, all unannotated tags were aligned with the reference genome. According to their genome positions and hairpin structures predicted by software Mireap_v0.2(https://sourceforge.net/projects/mireap/), the novel miRNA candidates were identified. Transcripts per million (TPM) was used to determine miRNA expression levels.

### Differentially expressed lncRNA, miRNA, and mRNA

Differentially expressed transcripts across samples or groups were identified as mRNA and lncRNA with a fold-change ≥2 and a false discovery rate (FDR) < 0.05, miRNAs with a fold-change ≥2 and *p* < 0.05, in comparison tosignificant DE lncRNA, DE miRNA, and DE mRNA by DESeq2 [26] or edgeR package [33].

### CeRNA network construction

Competitive endogenous RNAs (ceRNAs) are transcriptional regulators that modulate the expression of transcripts through competitive binding. The miRNA-mRNA and miRNA-lncRNA interactions were predicted using TargetScan and miRanda, and the competitive pairs of mRNA-lncRNA were identified using a hypergeometric test and Pearson’s correlation coefficient. Based on the sharing of miRNA binding sites and competitive pairs between lncRNAs and mRNAs, a ceRNA network was constructed as follows: *p* < 0.01, FDR < 0.01, and with the hypergeometric test [34–36]. Visualization of ceRNA was performed using the Cytoscape software (http://cytoscape.org/).

Spearman’s rank correlation coefficient between miRNA and candidate ceRNA was calculated for target gene pairs, and target gene pairs with correlation coefficients≤ − 0.7 were screened. The ceRNA theory believes that the expression levels of ceRNAs competing for the same miRNA are positively correlated. In turn, we calculated the Pearson correlation coefficient for the expression levels between the ceRNA pairs obtained in the previous step, and selected the ceRNA pairs with a correlation coefficient of more than 0.9. Based on the above threshold screening, and then using the hypergeometric distribution test [37], the ceRNA pairs with a *P* value < 0.05 were screened as the final ceRNA pairs.

### Expression analysis of DE lncRNA, DE miRNA, and DE genes using RT-qPCR

Primers were designed according to transcriptome sequencing data for CCs and GCs using Primer Premier 6.0. RT-qPCR was performed on 12 DEmRNAs,12 DE lncRNAs, and 8 DE miRNAs selected from RNA-seq data according to their latent functional importance. First-strand cDNA was synthesized using the Evo M-MLV RT Kit with gDNA clean for qPCR (Accurate Biotechnology, Hunan, China). RT-qPCR was performed on each sample in triplicate using the SYBR Green Premix Pro Taq HS qPCR Kit (Accurate Biotechnology, Hunan, China) on a LightCycler®480 Instrument II (Roche, Switzerland) in a 20 μL reaction volume. The cycling parameters for the PCR amplification were as follows: 95 °C for 30 s, followed by 40 cycles at 95 °C for 5 s and 60 °C for 30 s (Table 1, 2 and 3). Amplification specificity was evaluated using melting curve analysis. The relative expression of target gene transcripts was calculated using the comparative Ct method (2^-ΔΔCt^) and subjected to statistical analysis using SPSS software (version 19).

**Table 1:** RT-qPCR primers of mRNA for cumulus cells and Granulosa cells

Gene	Transcript ID	Primer sequences (5′-3′)	Product Size (bp)
RAC1	**ncbi_102273253**	**F: CCCAACACACCCATCATC** **R: TCGCCTCATCAAACACTG**	**202**
CDKN1A	**ncbi_102268672**	**F: ACTTGGACCTGTCGCTGT** **R:GGAGTGGTAGAAATCTGTCAT**	**131**
VEGFA	**ncbi_102270607**	**F: GGCTGCTGTAATGACGAA** **R: TCTCCTATGTGCTGGCTT**	**104**
ITGB1	**ncbi_102273972**	**F: AGACGACTTGGAGAATGTG** **R: GCTGGTGTTGTGCTAATG**	**135**
SGK1	**ncbi_102272039**	**F: AGTTGTTCTACCATCTCCAG** **R: CGTTGTGCCATTGTGTTC**	**215**
FGFR4	**ncbi_102267813**	**F:CCACCACATTGACTACTACA** **R: TCCAAACGACCACACATC**	**118**
JAK3	**ncbi_102286011**	**F: CGGATGATGGGATGTGAG** **R: AATGATGGTCGGTCTTGAG**	**158、**
NR4A1	**ncbi_102288141**	**F: GGAGGTGATTCGCAAGTG** **R: CCAGACGGAGGATAAAGAG**	**113**
ITGA6	**ncbi_102265941**	**F:AAGATGATATGGATGGAGGAG** **R: AACGCAATGTAATGGAAGTC**	**124**
LAMA5	**ncbi_102266736**	**F: GTGGTGTCCTTGGTGAAC** **R: GCTGATGTCCTTGATACTGT**	**195**
AKT3	**ncbi_102265427**	**F: GCCTTGGACTATCTACATTC** **R: CCCGCACATCATTTCATAC**	**255**
CCND1	**ncbi_102270546**	**F: CGAGGAGAACAAGCAGAT** **R: GCGGTGATAGGAGAGGAA**	**172**
β-actin	**NM_173979.3**	**F: CCGTGACATCAAGGAGAAG** **R: AGGAAGGAAGGCTGGAAG**	**174**

**Table 2:** RT-qPCR primers of LncRNA for cumulus cells and Granulosa cells

Types	Transcript ID	Primer sequences (5′-3′)
	MSTRG.17330.1	F: GAAGGCAGGAGAAGAAGG
LncRNA	(PHLPP1) XR_001351765.1(TEK)	R: GAACCAGGACCAGAATGAG F: CCTCTCTAAATACCTGGAACC R: AACACCTTACACGATAGCC
	MSTRG.1083.1 (PCGF6) MSTRG.8701.9 (MDM4) MSTRG.13933.1 (ID2) MSTRG.12026.1 (SMURF1) MSTRG.1083.1 (PCGF6) MSTRG.17464.1 (PRKAR1B) MSTRG.919.1 (CDKN2A) MSTRG.13216.1 (Pol) MSTRG.3682.1 (PAOX) MSTRG.9809.1 (LOC102277897)	F: GGCTTCTCTGGTGGTTCA R: AGGGTAGATGGGCTGGAA F: GACCTTGCTTGCTTGGAA R:CACAGACTTAGAGAACTTACAG F: TGTGCGGGATTACTTTGC R: AGGCTGACTGGACTCTGA F: CAAGACAGGAAGCCAGTG R: CAGATACAAACCCAGGAGTG F: GCAAGGAGAAGTGAGGATT R: CTGAGCGTGAAGAGGAAG F: ATGGCTGAACTCAAGTCC R: TGGGTTTCTCCTGTTGTG F: CGCTACGCTCCACTTCTA R: CACTTCAGGTTCTCACAGG F:GAGGAGAAGAGGAGGAGAG R: TGGCAGGAAGTCAGAGATA F: AAGAGATGGAAGGACTGG R: GACTGAACAAGGGCATTT F: TGCGACTTCACTTTCACTT R: CAGATACAGGAGCCAGGAT

**Table 3:** RT-qPCR primers of miRNA for cumulus cells and Granulosa cells

Types	Transcript ID	Primer sequences (5′-3′)
	miR-2431-y miR-7977-X	F: CACCCCCACTTGCATGACCCTGA F: TTCCCGGCCAACGCACCA
miRNA	miR-2284-Z miR-365-y miR-7862-y miR-342-y miR-195-X miR-574-X U6(XM_021614042.1)	F: AAAAAGTTCGTTTGGGTTTTCT F: TAATGCCCCTAAAAATCCTTAT F: TGGTGCTCCCTGGAGCTGAGC F:TCTCACACAGAAATCGCACCCATC F: TAGCAGCACAGAAATATTGGCA F: TGAGTGTGTGTGTGTGAGTGTGT F: GGAACGATACAGAGAAGATTAGC R: TGGAACGCTTCACGAATTTGCG

## Results

### Morphology of granulosa and cumulus cells

**Fig. 2:**
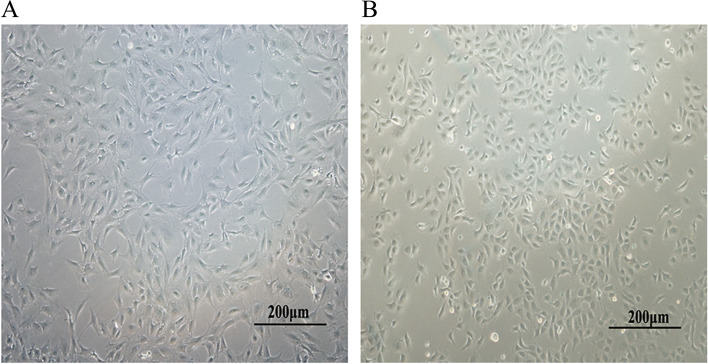
Primary culture of granulosa cells and cumulus cells (10× magnification). **A** Granulosa cells, **B** Cumulus cells

An inverted phase contrast microscope showed that the flat primary GCs displayed typical epithelioid-like growth and the cells had a clear nuclear boundary. The cells were polygonal or irregular polygonal stellate cells, tightly connected, intact, and in good condition (Fig. 2A). After 48 h of culturing primary CCs in vitro, they adhered to the wall and grew from a round shape to a long spindle shape, extending at both ends, and there were large gaps between the cells. When the cells grew to approximately 90% confluency, they became irregular polygons, with stable shapes, long spindles, and the occasional regular polygon (Fig. 2B).

### Overview of RNA-seq results in granulosa and cumulus cells

Six cDNA libraries, including three from the cumulus cell group (CC: CC-1, CC-2, and CC-3) and three from the granulosa cell group (GC: GC-1, GC-2, and GC-3), were constructed and analyzed by high-throughput sequencing. A total of 77.076 G clean bases were obtained and deposited in the National Center for Biotechnology Information database under accession number SRP307630. After quality control and filtering the raw reads, 512, 524, and 562 high-quality clean reads were generated from the six libraries. High-quality clean reads were compared to the ribosome of the species (mismatch number: 0) and to remove the reads corresponding to ribosomal RNA, and 511,825,690 effective reads were obtained. The average Q30 was 91.765% and the average GC content was 48.636%. Ribosomal RNA reads were filtered based on the updated reference genome of the yak [38] and the majority of effective reads were successfully mapped; the average mapped ratio was 92.34%. The differentially expressed gene (DEG) analysis based on the genome was reliable.

### Differential expression analysis of DE lncRNAs, DE mRNAs and DE miRNAs

**Fig. 3:**
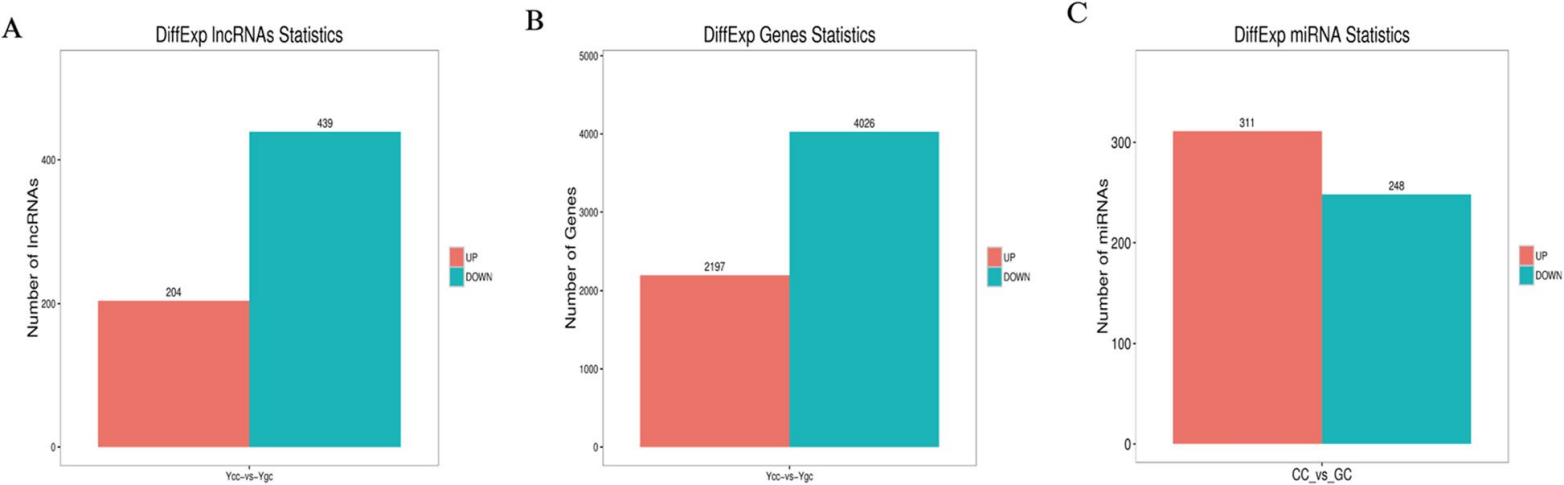
Different genes of upregulation and downregulation. **A** lncRNA, **B** mRNA, **C** miRNA

**Fig. 4:**
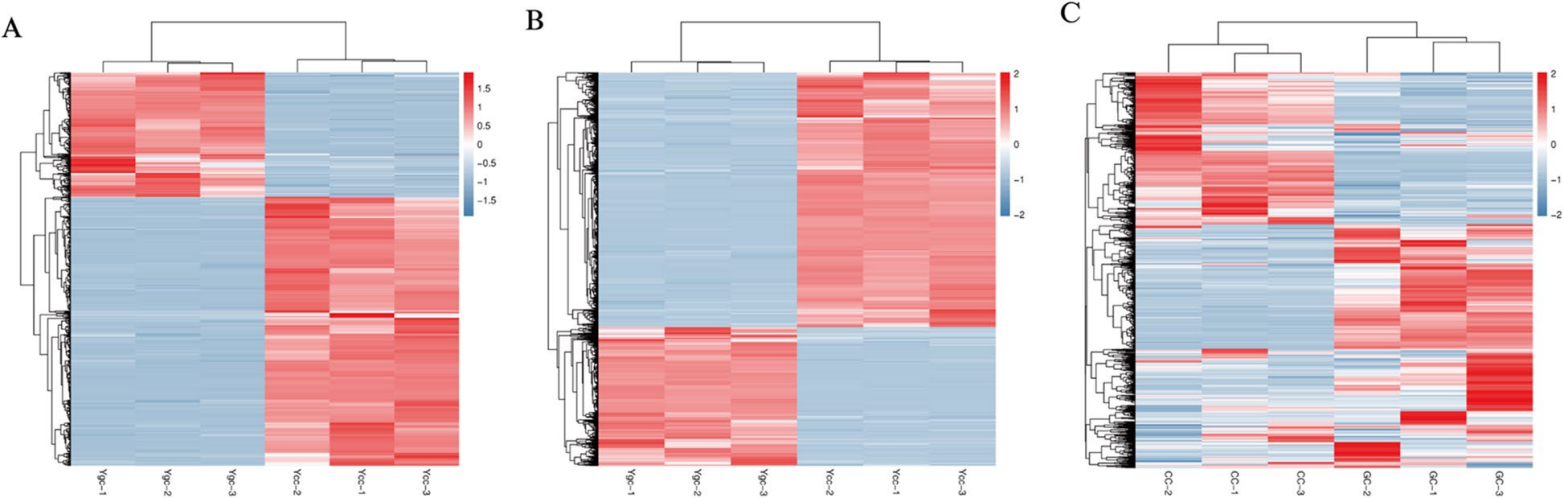
Each column represents a sample, and each row represents a lncRNA. The expression level of lncRNA in different samples is represented by different colors. The redder the color, the higher expression level, and the bluer the color, the lower the expression level. Gene expression number and profiling analyses of differentially expressed lncRNA and mRNA among CC and GC. (**A**) Heat map of the DE lncRNAs, (**B**) Heat map of the DE mRNAs, (**C**) Heat map of the DE miRNAs

A total of 1411 lncRNAs were obtained from the six libraries, and 643 significantly differentially expressed (DE) lncRNAs were identified, among which 204 DE lncRNAs were upregulated, and 439 DE lncRNAs were downregulated (Fig. 3A, Table S4). The total DE lncRNAs are presented as a heatmap based on gene expression (Fig. 4A). A total of 20,519 mRNAs and 6223 significantly differentially expressed mRNAs were identified, among which 2197 DE mRNAs were upregulated and 4026 DE mRNAs were downregulated (Fig. 3B, Table S5). Total DE mRNA is presented as a heat map based on gene expression (Fig. 4B). Additionally, a total of 2248 miRNAs and 559 DE miRNAs were identified by stringent thresholds (FDR < 0.05), among which 311 DE miRNAs were upregulated, and 248 DE miRNAs were significantly downregulated between the two groups (Fig. 3C, Table S6). The total DE miRNAs are presented in a heat map based on gene expression (Fig. 4C).

### Regulatory ceRNA network (DE lncRNA-DE miRNA-DE mRNA) of CCs and GCs

**Fig. 5:**
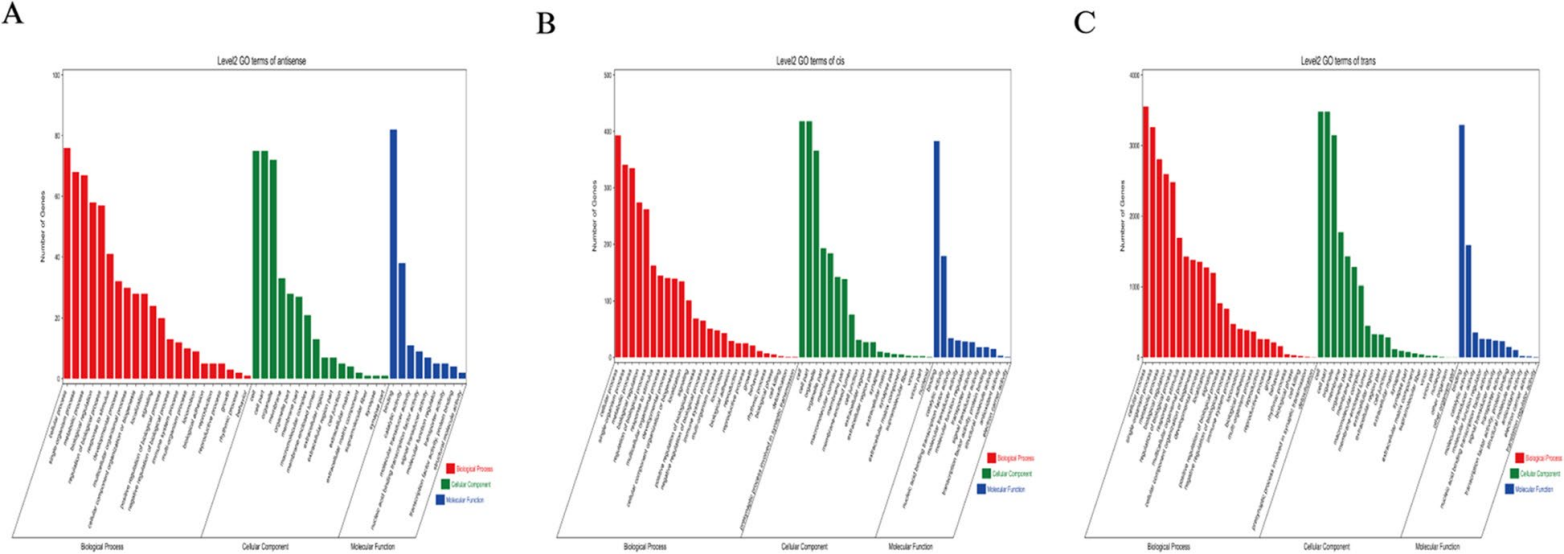
GO functional annotation histogram of the candidate genes related to DE lncRNAs and DE mRNAs

**Fig. 6:**
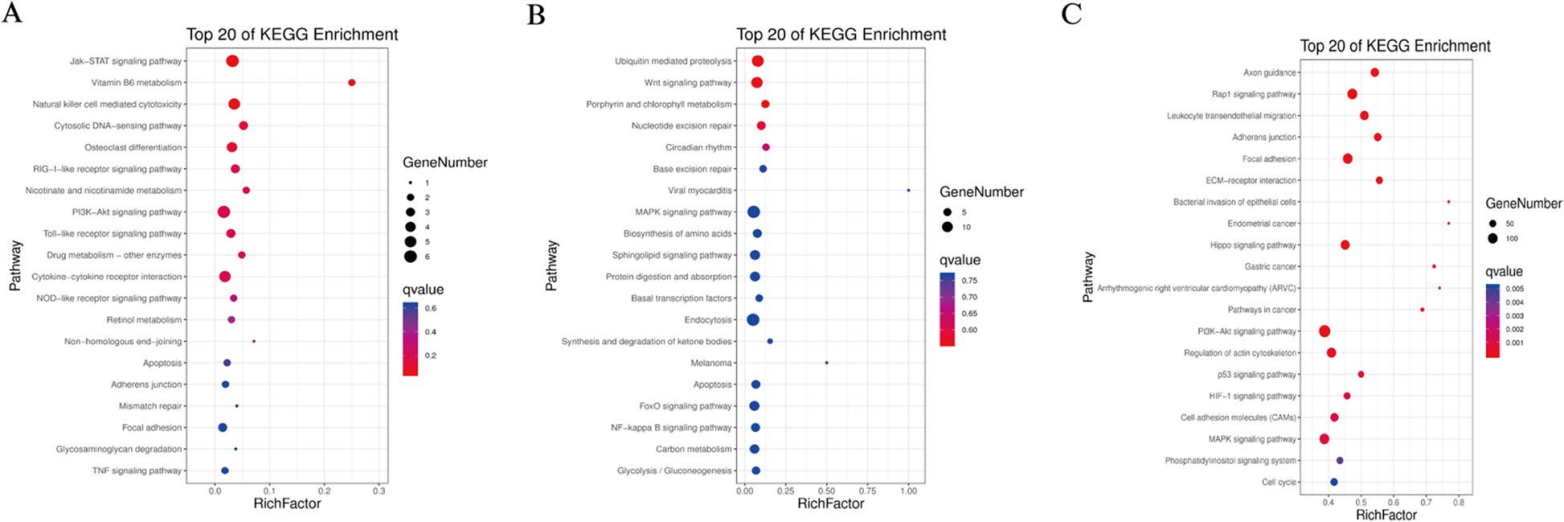
KEGG pathway assessment of the source genes of differential top 20 lncRNAs and mRNAs

**Fig. 7:**
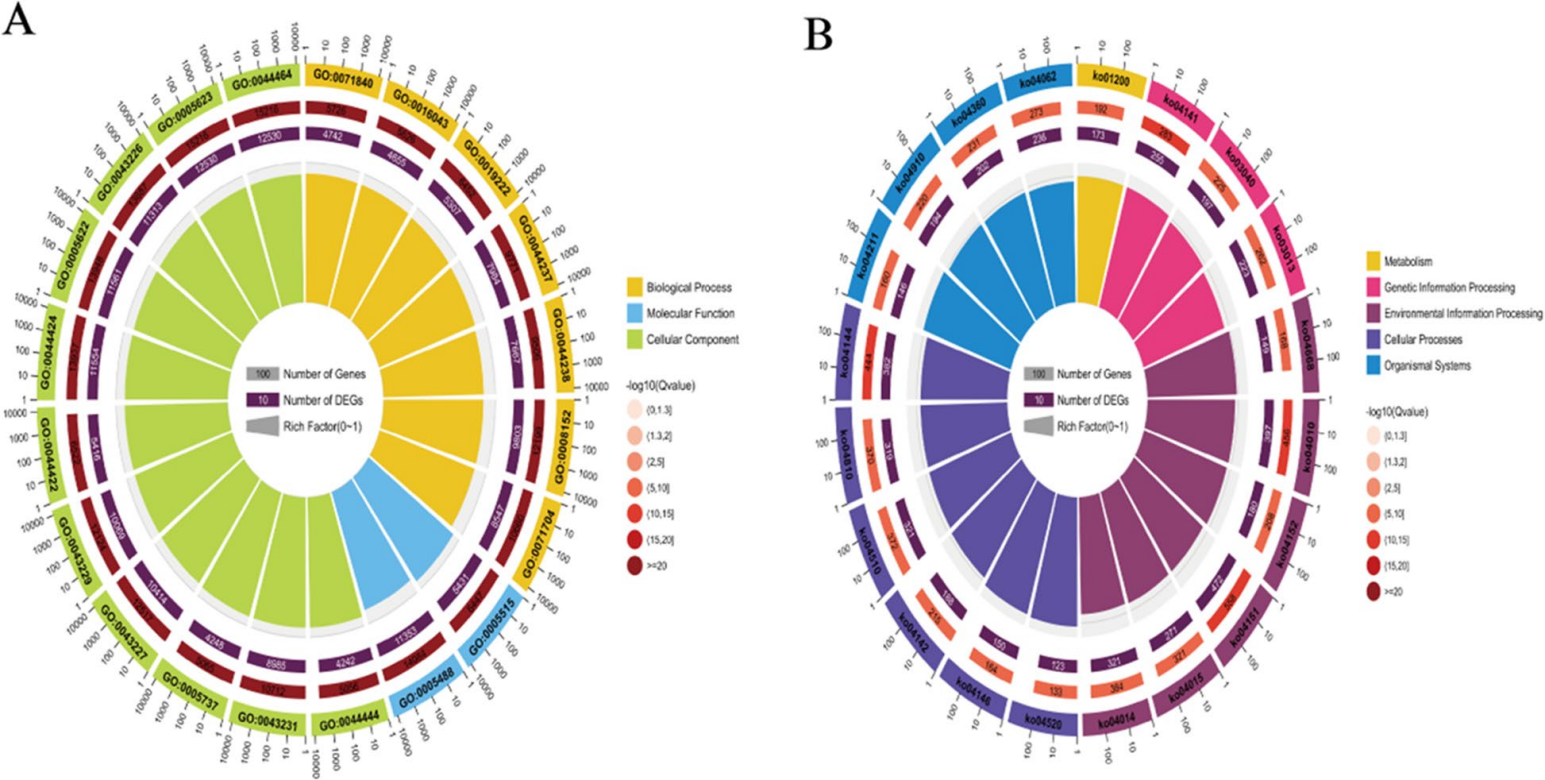
Radar map representations of the target genes of differential miRNAs. **A** GO functional annotation. **B** KEGG pathway assessment

We performed gene ontology (GO) enrichment and Kyoto Encyclopedia of Genes and Genomes (KEGG) enrichment analysis of DE mRNAs. GO enrichment analysis revealed that these DE mRNAs were significantly enriched in some target genes involved in cell adhesion, cell differentiation, regulation of developmental processes, cell proliferation, embryo development, regulation of signal transduction, programmed cell death, and aromatic compound biosynthetic process (Figs. 5,7A, Table S7). The ordinate represents a GO term, while abscissa represents the enrichment factor. The number of differences in this GO term is divided by all the numbers, with size indicating the number; the redder the color, the smaller the P/Q value. KEGG enrichment analysis showed that these target genes were significantly enriched in many pathways, including leukocyte trans-endothelial migration ECM-receptor interaction, MAPK signaling, Hippo signaling, cell cycle, cell adhesion, PI3K-Akt signaling, and regulation of the actin cytoskeleton (Figs. 6, 7B, Table S8). The X-axis indicates the detailed terms, while the Y-axis indicates the gene numbers.

**Fig. 8:**
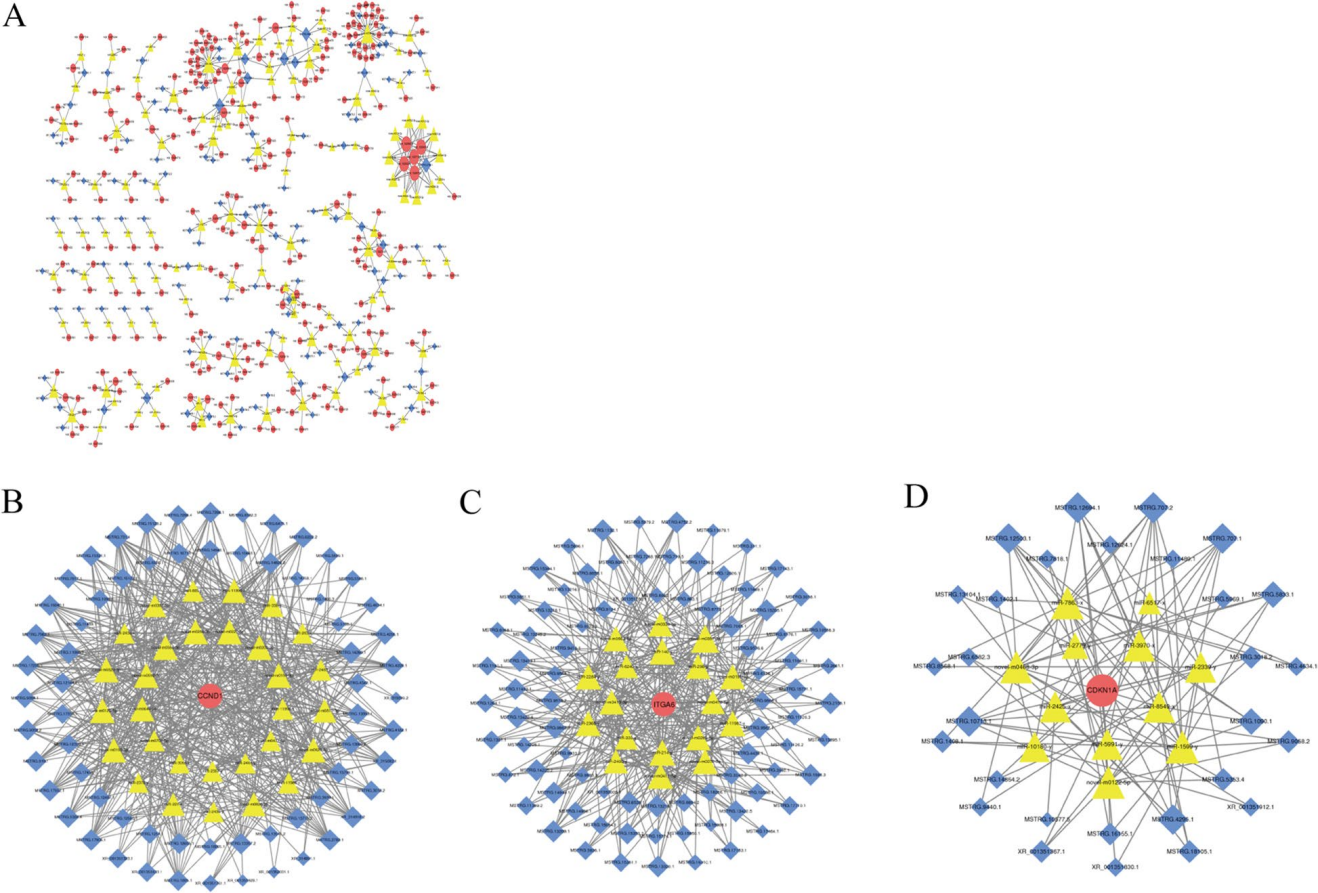
(**A**) CeRNA regulatory network in cumulus cells and granulosa cells in yak ovary, (**B**) Subnetwork of MSTRG.13544.2-miR-221-x|miR-2357-x|miR-338-y|novel-m0707-5p-ncbi_102270546 (CCND1), (**C**)MSTRG.9576.6-miR-11,987-x|miR-2284-y|novel-m0070-3p|novel-m0191–3p-ncbi_102265941 (ITGA6), (D) MSTRG.5969.1-novel-m0122-5p|novel-m0468-3p-ncbi_102268672 (CDKN1A)

We set the parameters according to the gradient method and found that the negative correlation was < − 0.95, the positive correlation was > 0.95, and when *P* < 0.01, there were a total of 540 relationship pairs. We constructed a mini-ceRNA (DE lncRNA-DE miRNA-DE mRNA) network of important RNAs (Fig. 8A, Table S9). Therefore, we constructed a ceRNA network in which the network diagram of mRNA-miRNA-lncRNA was drawn using Cytoscape, in which the mRNA is represented by circles, miRNA triangles, and lncRNA diamonds. During the analysis, according to the process parameters: negative correlation was ≤ − 0.7, positive correlation ≥0.9, and a *P* value < 0.05. There, were a total of 53,034 relationship pairs, and so many relationship pairs could not be presented through a network diagram.

Among them, three mRNAs with significant differences were screened, their related lncRNA and miRNA relationship pairs were selected, and a correlation network map was constructed. The subnetworks of ncbi_102270546 (CCND1) are displayed (Fig. 8B, Table S10), showing that CCND1 expression is regulated by 31 miRNAs and 66 lncRNAs. The most significant differences were observed in MSTRG.13544.2 and miR-221-x|miR-2357-x|miR-338-y|novel-m0707-5p. The subnetworks of ncbi_102265941 (ITGA6) and ncbi_102268672 (CDKN1A) are shown (Fig. 8C, D and Table S10), respectively. The target gene ITGA6 was regulated by 12 miRNAs and 84 lncRNAs, and the relationship pairs with significant differences were MSTRG.9576.6-miR-11,987-x|miR-2284-y|novel-m0070-3p|novel-m0191–3p-ncbi_102265941. In addition, CDKN1A was regulated by 12 miRNAs, 29 lncRNAs, and the differentially significant relationship pairs were MSTRG. 5969.1-novel-m0122-5p | novel-m5969-3p-ncbi102268672.

We identified 12 known mRNAs related to cell proliferation, differentiation, regulation of signal transduction and cell adhesion, cell growth, death, and regulation of cyclin. Some target genes are involved in cell adhesion, cell differentiation, regulation of developmental processes, cell proliferation, embryo development, signal transduction, programmed cell death, and aromatic compound biosynthetic processes. These RNAs are involved in many pathways, including leukocyte trans-endothelial migration, ECM-receptor interaction, the MAPK signaling pathway, the Hippo signaling pathway, the cell cycle, cell adhesion, the PI3K-Akt signaling pathway, and regulation of the actin cytoskeleton.

The diamond: lncRNAs. The triangles: miRNAs. The circles: mRNAs.

### Validation of DE lncRNA, DE miRNA and DE mRNA expression by RT-qPCR

**Fig. 9:**
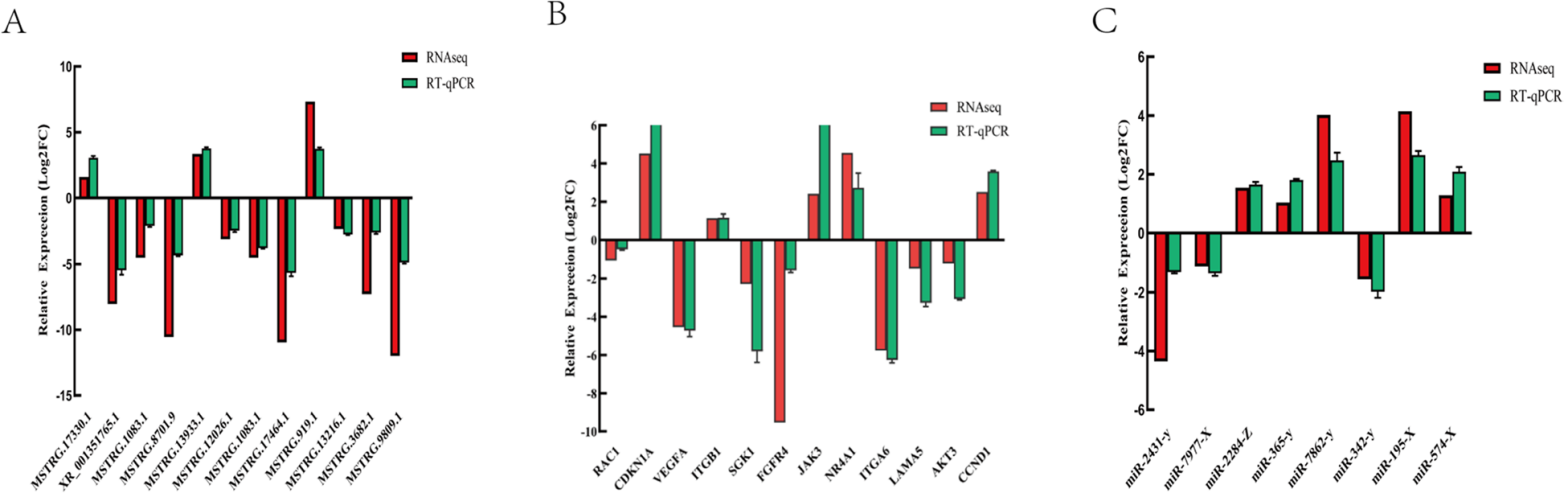
Comparison of the gene expression levels determined by RNA-seq and RT-qPCR. **A** DE lncRNAs**, B** DE mRNAs**, C** DE miRNAs

The relative expression (log2FC) of these DEGs was similar between the two approaches, although some quantitative differences were found between the RT-qPCR and RNA-seq analytical platforms (Fig. 9). Therefore, the RNA-seq results were reliable and could be used for bioinformatic analysis.

## Discussion

The ovary is the female gonad present as a pair in most animals and is responsible for the generation of female gametes and the production of hormones that regulate reproductive functions. The growth and development of mammalian oocytes occurs in a specialized compartment of the ovary called the follicle. Follicle development in mammals mainly includes primordial, primary, secondary, tertiary, and mature follicle stages. Primordial follicles are composed of a single flat layer of GCs surrounded by immature oocytes. Further differentiation of primary follicles into preantral follicles involves acquisition of a theca layer around the GC layer and GC division through mitosis to form multiple layers of GCs (the theca layer remains as a single layer at this stage). The growth and development of oocytes affect the functional activities of surrounding somatic cells [39]. The interaction between oocytes and surrounding somatic cells progresses as the oocyte is released from the state of quiescence toward ovulation, fertilization, and zygote formation. As follicles grow and cavities form, somatic cells are divided into two distinct subtypes: the CCs, which surround and are in close metabolic contact with the oocyte, and the parietal GCs, which are follicle-forming cells [40]. Numerous factors affect oocyte developmental capacity and granulosa cell and follicular microenvironment changes, including heat stress [41].GCs are tightly connected to oocytes in mammalian ovaries through gap junctions and provide oocytes with the essential signals and metabolites required for oocyte growth and maturation [42].CCs support meiotic arrest and cytoplasmic maturation of the oocyte by exporting cyclic adenosine nucleotides [43], calcium [44], other metabolites [45], and unknown signals that control transcription in the occluded oocyte [46].

Proper folliculogenesis and ovulation can only be achieved through reciprocal signaling events generated by intimate granulosa cell-oocyte communication [47]. Before ovulation, differentiated GCs can secrete a large amount of sex hormones and growth factors in follicles to ensure successful ovulation [48]. The functions of the two different cells in the whole follicle development process are not consistent: CCs play a key role in the normal growth and development of oocytes, while GCs mainly play an endocrine function to support the growth of the follicle [8]. After ovulation, GCs undergo terminal differentiation into luteal cells and continue to play an endocrine role, while CCs can co-ovulate with oocytes to assist in egg retrieval and the sperm acrosome reaction. Although some functional differences between these two types of follicular somatic cells have been well described, it is not clear which genes regulate their developmental pathways. Previous studies have found that the ability of oocytes to promote follicular cell proliferation was first directly demonstrated in mice [49].

GC proliferation, steroid production, and morphological changes are primarily linked to molecular pathways. Sheep GCs overexpress the sonic hedgehog (Shh) signaling pathway, which has the same potential role as the Hedgehog pathway in increasing GC proliferation at the preluminal stage [50]. An important factor in maintaining follicle and oocyte growth for reproductive success is proper regulation of GC activity [51]. In the ovary, the β-catenin-dependent canonical pathway of WNT4 leads to an ovarian-dependent pathway. WNT proteins can also signal through Rho-GTPases, utilizing non-canonical pathways associated with changes in polarized cell shape and migration [52]. This ameliorates small changes in cell shape that affect GCs during early folliculogenesis. The study uncovered two classes of cellular interactions, demonstrating a complex dialogue between compartments: molecular dialogue (signaling pathways) and physical communication (gap junctions and trans-regional projections) [53].

LncRNAs are more than 200 nucleotides in length and constitute a family of transcripts that are unable to encode protein [54]. This theory suggests that lncRNAs act as natural sponges or decoys to competitively bind certain miRNAs and reduce the binding of miRNAs to corresponding target genes, resulting in altered miRNA target gene expression [55, 56]. However, it remains unclear whether abnormal lncRNAs exert ceRNA effects on some miRNAs and exert certain effects on signal transduction during cell proliferation and differentiation by indirectly regulating the expression of target mRNAs.

In this study, a total of 1411 lncRNAs and 643 significantly DE lncRNAs were obtained from the six libraries, of which 204 DE lncRNAs were upregulated and 439 DE lncRNAs were downregulated. A total of 20,519 mRNAs and 6223 significantly differentially expressed mRNAs were identified, among which 2197 DE mRNAs were upregulated and 4026 DE mRNA were downregulated. Additionally, a total of 2248 miRNAs and 559 significantly differentially expressed miRNAs were identified, among which 311 DE miRNAs were upregulated, and 248 DE miRNAs were downregulated significantly between the two groups. Some target genes are involved in cell adhesion, cell differentiation, regulation of developmental processes, cell proliferation, embryo development, signal transduction, programmed cell death, and aromatic compound biosynthetic processes. These RNAs were involved in many processes and pathways, including leukocyte trans-endothelial migration, ECM-receptor interaction, the MAPK signaling pathway, the Hippo signaling pathway, the cell cycle, cell adhesion, the PI3K-Akt signaling pathway, and regulation of the actin cytoskeleton. Few lncRNAs showed clear spatiotemporal expression and specificity in the process of tissue growth and differentiation.

Cyclin D1 is encoded by the CCND1 gene located on chromosome band 11q13 and promotes cell cycle progression during the G1-S phase [57]. Activation of the PI3K/Akt pathway promotes an up-regulation mechanism of CCND1, in which mTor can activate CCND1 translation and promote the synthesis of cyclin D1/CDK4 and CDK6 complexes involved in cell cycle progression [58]. Amplification of CCND1 is prevalent in human cancers. Dai et al. propose a role for CCND1 in promoting ovarian cancer cell proliferation, which can be alleviated by treatment [59]. CDKN1A encodes p21 and is a cyclin-dependent kinase (Cdk) inhibitor [60]. In cancer cell lines, p21 acts as an activator to synthesize and activate cyclin D/Cdk4 or Cdk6 complexes to enhance proliferation efficiency [61]. Hyperphosphorylated p21 activates Cdk1 upon G2/M transition [62]. p21 is both an inhibitor and activator of the cell cycle, depending on the cellular environment and its expression levels. In contrast, p21 is involved in checkpoint control and initiates temporary cell cycle arrest [63].. Interestingly, we also enriched the network graph for ITGA6. When ITGA6 forms α6β1 and α6β4 integrin complexes with other integrin subunits, embryogenesis, organogenesis, and cancer cell invasion can be mediated [64]. From these differential genes, we will provide more predictive target genes for the differentiation of GCs and CCs.

Little research has been conducted to explore the mechanism of lncRNAs in GCs and CCs. The present study had several strengths. We constructed a ceRNA network in which a network diagram of mRNA-miRNA-lncRNA was drawn. There were 540 relationship pairs, which provided a reference for exploring biological processes such as proliferation, differentiation, and signal transduction between CCs and GCs. The evidence we provide here suggests that differential mRNA-miRNA-lncRNA may play an important role in follicle development and ovulation. However, the specific mechanism requires further exploration in cell and animal experiments.

## Conclusions

In conclusion, this study systematically characterized the ceRNA regulatory network and biochemical parameters of CCs and GCs. Many identified lncRNAs, miRNAs, and mRNAs provide a reference for further research on the regulatory mechanisms of ceRNAs. Our findings also provide new insights into the molecular mechanisms of CC and GC proliferation, differentiation, and signal transduction, which will help explore the effects of CCs and GCs on follicle growth and oocyte development.

### Institutional review board statement

The experimental protocol was reviewed and approved by the Faculty of Animal Veterinary Medicine, Gansu Agricultural University (approval number GSAU-Eth-VMC-2022-018) and performed in agreement with the Care and Use Guidelines of Experimental Animals published by the Ministry of Science and Technology of the People’s Republic of China.

## Supplementary Information

**Additional file 1: Table S1.** RT-qPCR primers of mRNA for cumulus cells and Granulosa cells. **Table S2.** RT-qPCR primers of LncRNA for cumulus cells and Granulosa cells. **Table S3.** RT-qPCR primers of miRNA for cumulus cells and Granulosa cells. **Table S4.** DE lncRNAs in cumulus cells and granulosa cells in yak ovary.** Table S5.** DE mRNAs in cumulus cells and granulosa cells in yak ovary. **Table S6.** DE miRNAs in cumulus cells and granulosa cells in yak ovary. **Table S7.** GO miRNAs in cumulus cells and granulosa cells in yak ovary. **Table S8.** KEGG miRNAs in cumulus cells and granulosa cells in yak ovary. **Table S9.** ceRNA regulatory network in cumulus cells and granulosa cells in yak ovary. **Table S10.** ceRNA regulatory network of differential genes CCND1, ITGA1, and CDKN1A in cumulus cells and granulosa cells in yak ovary.

## Data Availability

Raw sequencing reads of lncRNA-seq and miRNA-seq in this paper have been deposited in the NCBI Sequence Read Archive (SRA) under BioProject accession number PRJNA704089.

## Abbreviations

CCs: Cumulus cells
GCs: Granulosa cells
GO: Gene Ontogeny
RT-qPCR: Quantitative real-time PCR
KEGG: Kyoto Encyclopedia of Genes and Genomes
DMEM: Dulbecco’s modified Eagle’s medium
FBS: Foetal bovine serum
PBS: Phosphate buffered saline
