# Supplementary material for: Integrated analysis of the expression profiles of the lncRNA-miRNA-mRNA ceRNA network in granulosa and cumulus cells from yak ovaries
Source: BMC Genomics. 2022 Sep 3;23:633. doi: 10.1186/s12864-022-08848-3 (PMC9441039; doi:10.1186/s12864-022-08848-3)
Supplement: Supplementary file 1 — Additional file 1: Table S1. RT-qPCR primers of mRNA for cumulus cells and Granulosa cells. Table S2. RT-qPCR primers of LncRNA for cumulus cells and Granulosa cells. Table S3. RT-qPCR primers of miRNA for cumulus cells and Granulosa cells. Table S4. DE lncRNAs in cumulus cells and granulosa cells in yak ovary. Table S5. DE mRNAs in cumulus cells and granulosa cells in yak ovary. Table S6. DE miRNAs in cumulus cells and granulosa cells in yak ovary. Table S7. GO miRNAs in cumulus cells and granulosa cells in yak ovary. Table S8. KEGG miRNAs in cumulus cells and granulosa cells in yak ovary. Table S9. ceRNA regulatory network in cumulus cells and granulosa cells in yak ovary. Table S10. ceRNA regulatory network of differential genes CCND1, ITGA1, and CDKN1A in cumulus cells and granulosa cells in yak ovary. [file 12864_2022_8848_MOESM1_ESM.zip › Supplementary Materials/Table-S1-3 RT-qPCR primers of DElncRNA, DE miRNA and DE mRNA in yak ovary..docx]

Table 1. RT-qPCR primers of mRNA for cumulus cells and Granulosa cells

| Gene | Transcript ID | Primer sequences (5'-3') | Product Size(bp) |
| --- | --- | --- | --- |
| RAC1 | ncbi_102273253 | F: CCCAACACACCCATCATC  R: TCGCCTCATCAAACACTG | 202 |
| CDKN1A  VEGFA  ITGB1  SGK1  FGFR4  JAK3  NR4A1  ITGA6   LAMA5  AKT3  CCND1 | ncbi_102268672  ncbi_102270607  ncbi_102273972  ncbi_102272039  ncbi_102267813  ncbi_102286011  ncbi_102288141  ncbi_102265941  ncbi_102266736  ncbi_102265427  ncbi_102270546 | F: ACTTGGACCTGTCGCTGT  R:GGAGTGGTAGAAATCTGTCAT  F: GGCTGCTGTAATGACGAA  R: TCTCCTATGTGCTGGCTT  F: AGACGACTTGGAGAATGTG  R: GCTGGTGTTGTGCTAATG  F: AGTTGTTCTACCATCTCCAG  R: CGTTGTGCCATTGTGTTC  F:CCACCACATTGACTACTACA  R: TCCAAACGACCACACATC  F: CGGATGATGGGATGTGAG  R: AATGATGGTCGGTCTTGAG  F: GGAGGTGATTCGCAAGTG  R: CCAGACGGAGGATAAAGAG  F:AAGATGATATGGATGGAGGAG  R: AACGCAATGTAATGGAAGTC  F: GTGGTGTCCTTGGTGAAC  R: GCTGATGTCCTTGATACTGT  F: GCCTTGGACTATCTACATTC  R: CCCGCACATCATTTCATAC  F: CGAGGAGAACAAGCAGAT  R: GCGGTGATAGGAGAGGAA | 131  104  135  215  118  158、  113  124  195  255  172 |
| β-actin | NM_173979.3 | F: CCGTGACATCAAGGAGAAG  R: AGGAAGGAAGGCTGGAAG | 174 |

Table 2. RT-qPCR primers of LncRNA for cumulus cells and Granulosa cells

| Types | Transcript ID | Primer sequences (5'-3') |  |
| --- | --- | --- | --- |
|  | MSTRG.17330.1 | F: GAAGGCAGGAGAAGAAGG |  |
| LncRNA | (PHLPP1)  XR_001351765.1(TEK)  MSTRG.1083.1  (PCGF6)  MSTRG.8701.9  (MDM4)  MSTRG.13933.1  (ID2)  MSTRG.12026.1  (SMURF1)  MSTRG.1083.1  (PCGF6)  MSTRG.17464.1  (PRKAR1B)  MSTRG.919.1  (CDKN2A)  MSTRG.13216.1  (Pol)  MSTRG.3682.1  (PAOX)  MSTRG.9809.1  (LOC102277897) | R: GAACCAGGACCAGAATGAG  F: CCTCTCTAAATACCTGGAACC  R: AACACCTTACACGATAGCC  F: GGCTTCTCTGGTGGTTCA  R: AGGGTAGATGGGCTGGAA  F: GACCTTGCTTGCTTGGAA  R:CACAGACTTAGAGAACTTACAG  F: TGTGCGGGATTACTTTGC  R: AGGCTGACTGGACTCTGA  F: CAAGACAGGAAGCCAGTG  R: CAGATACAAACCCAGGAGTG  F: GCAAGGAGAAGTGAGGATT  R: CTGAGCGTGAAGAGGAAG  F: ATGGCTGAACTCAAGTCC  R: TGGGTTTCTCCTGTTGTG  F: CGCTACGCTCCACTTCTA  R: CACTTCAGGTTCTCACAGG  F:GAGGAGAAGAGGAGGAGAG  R: TGGCAGGAAGTCAGAGATA  F: AAGAGATGGAAGGACTGG  R: GACTGAACAAGGGCATTT  F: TGCGACTTCACTTTCACTT  R: CAGATACAGGAGCCAGGAT |  |

Table 3. RT-qPCR primers of miRNA for cumulus cells and Granulosa cells

| Types | Transcript ID | Primer sequences (5'-3') |
| --- | --- | --- |
|  | miR-2431-y  miR-7977-X | F: CACCCCCACTTGCATGACCCTGA  F: TTCCCGGCCAACGCACCA |
| miRNA | miR-2284-Z  miR-365-y  miR-7862-y  miR-342-y  miR-195-X  miR-574-X  U6(XM_021614042.1) | F: AAAAAGTTCGTTTGGGTTTTCT  F: TAATGCCCCTAAAAATCCTTAT  F: TGGTGCTCCCTGGAGCTGAGC  F:TCTCACACAGAAATCGCACCCATC  F: TAGCAGCACAGAAATATTGGCA  F: TGAGTGTGTGTGTGTGAGTGTGT  F: GGAACGATACAGAGAAGATTAGC  R: TGGAACGCTTCACGAATTTGCG |
